# Incidental intraoperative discovery of a pancreatic neuroendocrine tumor associated with chronic pancreatitis

**DOI:** 10.1186/1746-1596-7-132

**Published:** 2012-09-29

**Authors:** Valeriu Surlin, Sandu Ramboiu, Mirela Ghilusi, Iancu Emil Plesea

**Affiliations:** 1 Department of Surgery, University of Medicine and Pharmacy of Craiova, Petru Rares 2, 200393, Craiova, Romania; 2 Department of Pathology, University of Medicine and Pharmacy of Craiova, Petru Rares 2, 200349, Craiova, Romania

**Keywords:** Pancreatic neuroendocrine tumor, Chronic pancreatitis, Splenopancreatectomy

## Abstract

Pancreatic neuroendocrine tumors are a rare entity with an incidence between 2 per million to 5 per 100 000. Association with pancreatitis (acute or chronic) is rare and is considered to be determined by the tumoral obstruction of pancreatic ducts, but sometimes occurs without any apparent relationship between them. Non-functional neuroendocrine pancreatic tumors are usually diagnosed when either very large or metastatic. Small ones are occasionally diagnosed when imagery is performed for other diagnostic reasons. Intraoperative discovery is even rarer and poses problems of differential diagnosis with other pancreatic tumors. Association with chronic pancreatitis is rare and usually due to pancreatic duct obstruction by the tumor. We describe the case of a patient with a small non-functioning neuroendocrine tumor in the pancreatic tail accidentally discovered during surgery for delayed traumatic splenic rupture associated with chronic alcoholic pancreatitis. The tumor of 1.5cm size was well differentiated and confined to the pancreas, and was resected by a distal splenopancreatectomy.

**Conclusions:**

Surgeons should be well aware of the rare possibility of a non-functional neuroendocrine tumor in the pancreas, associated with chronic pancreatitis, surgical resection being the optimal treatment for cure. Histopathology is of utmost importance to establish the correct diagnosis, grade of differentiation, malignancy and prognosis.

**Virtual slides:**

The virtual slide(s) for this article can be found here: http://www.diagnosticpathology.diagnomx.eu/vs/2114470176676003.

## Background

Pancreatic neuroendocrine tumors are a rare entity, part of a larger group called gastroenteropancreatic endocrine tumors, with histologic origins in the neuroendocrine cells inside the pancreas [1].

Incidence ranges from 1–2 cases per million individuals [2] to 2.5–5 cases per 100000 [3] and it will probably grow more due to availability of modern and powerful diagnostic tools. Incidence in autopsies ranges between 1.6% and 10% [4].

Association with pancreatitis (acute or chronic) is rare and is considered to be determined by the tumoral obstruction of pancreatic ducts, but sometimes occurs without any apparent relationship between them [5].

### Case presentation

A 58 years old patient was admitted in our surgical department for faintness and dizziness after a fall from standing occurring a week before. Known as a heavy alcohol consumer, he was frequently brought in hospital because of staggering in the streets. He denied any personal medical or surgical antecedents and couldn't indicate any family medical history. He worked as farmer all his life and had a history of smoking around 20 cigarettes per day for 10 years but stopped 8 years ago. Physical examination was normal, laboratory data within normal range excepting a mild anemia. Abdominal ultrasonography diagnosed a 100/25 mm subcapsular hematoma on the posterior side and a 10 mm thick fluid collection on the medial side of the spleen. There was no free fluid, nor other lesion of parenchymal viscera. The initial decision was for conservative management, patient’s status improving in the following days. Third day after admission, the patient presented diffuse abdominal pain, cold sweats, tachycardia over 100/min and falling blood pressure. There was a strong suspicion of delayed splenic rupture and the patient was immediately taken to operating room.

At laparotomy there were free fresh and old blood clots in the peritoneal cavity and around the spleen, in quantity estimated up to 700–800 ml and a ruptured subcapsular hematoma on the posterior side of the spleen with active bleeding from an irregular-shaped mid-splenic fracture of 3 cm depth. No other lesions were found. Dissection of splenic vessels for vascular control, revealed a chronic pancreatitis with peripancreatic fibrosis and a a 1.5-2 cm tumor palpated in the pancreatic tail right in front of the splenic hilum. The tumor had a slightly more consistency than the pancreas. There were no enlarged lymphnodes around in this area. A splenopancreatectomy was decided then with a margin of at least 3 cm proximally. The patient had an uneventful postoperative course.

Gross and microscopic examination revealed, in the pancreatic fragment attached to the spleen, a 1.5 cm nodular, well circumscribed, firm, grayish tumor, unhomogenous, with hemmorhagic foci on section.

**Figure 1:**
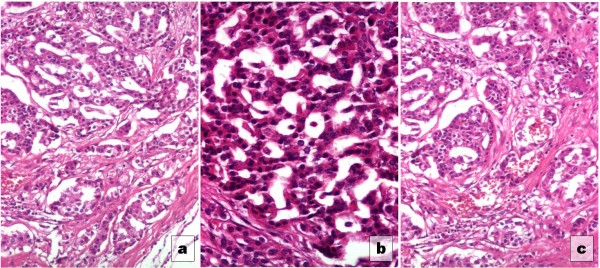
Histological profile of the studied tumor.

Microscopic examination revealed a tumor incompletely delineated by a thin fibrous capsule (see Figure 1a). Tumor cells presented a pattern predominantly pseudoglandular, consisting of polygonal cells with moderate eosinophilic cytoplasm and round-oval nuclei with relatively uniform shape and size (see Figure 1b). Small hemorrhagic foci were observed in the tumor (see Figure 1c). There was no vascular or perineural invasion, nor necrosis. Number of mitosis observed was low: 2–3 mitosis /10 high power fields (400x).

Figure 1 - (a) Neuroendocrine pancreatic tumor (adenocarcinoma) well delineated by a pseudocapsule, hematoxylin eosin (100x); (b) - Pseudoglandular pattern, hematoxylin eosin (200x); (c) - Haemmorhagic foci and variable fibro-collagen stroma, hematoxylin eosin (100x).

**Figure 2:**
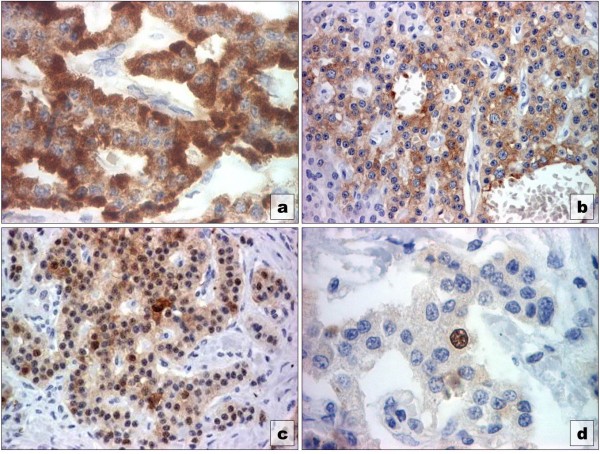
Immunohistochemical profile of the studied tumor.

Immunohistochemical stainings revealed tumor cells positive for chromogranin, synaptophysin, and neuron specific enolase (see Figure 2a, 2b, 2c). Proliferation markers as MIB-1 (directed against the Ki-67 antigen), were expressed in less than 2% of the tumor cells nuclei (see Figure 2d).

Figure 2 - (a) Intense and diffuse immunostaining of the tumor cells cytoplasm for Chromogranin, LSAB technique (400x); (b) – Diffuse immunostaining of the tumor cells cytoplasm for Synaptophysin, LSAB technique (200x); (c) – Immunostaining of the tumor cells cytoplasm for NSE, LSAB technique (200x); (d) - Rare Ki-67 positive neoplastic cells, LSAB technique, (400x).

Based on those characteristics (proliferating tumor confined to the pancreatic tissue, reduced size, and low proliferation index, absence of necrosis, no vascular or perineural invasion) the tumor was diagnosed as well differentiated with benign behavior according to the WHO classification [6]. 8 lymph nodes were examined and, apart from the presence of different degrees of histiocytosis, no tumoral involvement was found.

## Discussion

Generally there are two categories of pancreatic neuroendocrine tumors: functional, releasing in the peripheral blood active hormones responsible for a specific clinic syndrome and non-functional, secreting either a functionally inert hormone or in very small amounts, therefore not producing any clinical symptoms [7,8].

The proportion of non-functional tumors varies largely in published series. A review by Ito et al. found 48% of pancreatic neuroendocrine tumors as non-functional [9]. Kazanjian et al., in a series of 70 consecutive resected neuroendocrine tumors of pancreas found 71% of them non-functional [10].

The peak incidence for these tumors is between 30 and 60 years of age [11]. Early clinical diagnosis of non-functional pancreatic tumors is very rare because usually they become symptomatic only when they grow larger or metastasize. Otherwise they are discovered incidentally on the occasion of imagistic tests performed for other medical reasons. In our case, there is a very rare situation of intraoperative discovery.

Abdominal ultrasound was performed before surgery but, on regular examination, PNETs present a hypoechoic aspect that cannot be differentiated from other types of tumor, and could escape detection because of operator's inexperience, flatulence, obesity, deep situations and locations in the tail [12]. In the case we describe the old and organizing blood clots around the spleen surely contributed to hide the tumor.

Intraoperative discovery poses problems of diagnostic and therapeutic decision. Association with a grossly aspect of chronic pancreatitis raises the suspicion of pancreatic cancer. Pancreatic neuroendocrine tumors represents less than 3% from pancreatic tumors [13,14]. It is far more rare than pancreatic adenocarcinoma, with a ratio of 1:25 [6] being substantially rarer than adenocarcinomas and carrying a better prognosis [8]. Most neuroendocrine tumors of pancreas are located in the pancreatic head [15]. In our case the tumor was located near the splenic hilum.

In front of an accidentally discovered pancreatic tumor we had to decide whether to perform or not a frozen biopsy. Due to the fact that the tumor was well confined to the pancreas and relatively easy amenable to resection, there were no enlarged lymphnodes in the vicinity and the pancreas presented a firm consistency, well suitable for suture with low-risk for pancreatic fistula, a resection with safe margins was decided.

We think that in such cases a frozen section could not have brought any elements to change our decision. In neuroendocrine tumors of pancreas the only reason to perform a frozen section is when an enucleation is planned but even in such situations there could be problems of differential diagnosis with papillary tumors [16].

The mainstay of treatment for neuroendocrine tumors of pancreas is surgical resection. However, particularly for non-functional tumors, it remains controversial as to whether resection alters their natural history. Tumor enucleation can be performed in cases in which the pancreatic endocrine tumor is single, capsulated, of limited size (less than 2 to 3 cm) and peripheral, situated at a distance from the main duct [13,14]. The safe margin of resection is not clearly indicated in the litterature. If the tumor is macroscopically well delimited the margin can be very narrow, from few millimeters to 1 cm [16].

Immunohistochemistry is of outmost importance for the study of neuroendocrine tumors of pancreas. Several antibodies are available against neuroendocrine markers such as NSE, CD56, synaptophysin, CgA, and other hormones. It is important to discriminate well-differentiated forms from poorly differentiated carcinomas using malignancy markers [17]. The malignant or metastatic potential of neuroendocrine pancreatic tumors can be estimated using the tumor size, mitotic index, expression of KI-67 protein, vascular invasion, and perineural invasion [18]. The proliferation marker MIB-1 (directed against the Ki-67 antigen) helps to determine tumor grade and prognosis [3].

Association with pancreatitis (acute or chronic) was already reported in the literature. Mostly, there are reports of acute pancreatitis generated by obstruction of the main duct by the tumor [5,19,20]. In some of the cases the acute pancreatitis became chronic. In other, there was no evident relationship between chronic pancreatitis and the neuroendocrine tumor [5]. In 71% of the cases the tumor was malignant [19].

In our case the association between chronic pancreatitis and PNET seemed purely incidental because the pancreatitis involved grossly the entire pancreatic gland and the tumor was located in the proximity of the splenic hilum. In addition, the patient was a chronic alcohol consumer, a well-known etiological factor for chronic pancreatitis.

There are very few studies in the literature searching a common genetic pathway for chronic pancreatitis and pancreatic neuroendocrine tumors. In an interesting comparative study in search for specific molecular markers, Bloomstone et al. [21] compared them with different other pancreatic diseases as chronic pancreatitis and pancreatic adenocarcinomas. They found that ELOVL4 (a gene codifing the protein for the elongation of very-long-chain fatty acids 4-like, responsible for the biosynthesis of fatty acids) and CALCR (gene responsible for calcitonine receptor) were increased both in neuroendocrine tumors and chronic pancreatitis.

Overall prognosis after resection is much better than of other pancreatic tumors. In a review of 3851 cases, survival was 59.3% at 5 years and 37.7% at 10-years. Age, grade, distant metastases, tumor functionality, and type of resection were independent predictors of survival, meanwhile gender, race, socioeconomic status, tumor size, nodal status, margins, adjuvant chemotherapy, and hospital volume had no influence [22].

Beside association with chronic pancreatitis, there are reports of a unique association between carcinoid tumors and renal cell carcinoma in a mature cystic teratoma of a horseshoe kidney. In this case the tumor presented also a low grade of malignancy and differentiation as adenocarcinoma as in our case [23]. Primary renal carcinoid tumors are morphologically and histologically similar to those in other sited. The surgical resection is curative and prognosis is good in the absence of metastasis but long term follow-up is recommended because distant recurrence may occur up to seven years postoperatively [23].

Immunohistochemical markers may be related also with prognosis beside their diagnostic role. Expression of NSE and CD 56 was studied in renal cell carcinoma and was correlated with prognosis. First of all, NSE marker was exhibited by 48% of the renal cell carcinoma in a series of 152 cases. In the same series there was an 18% of CD56 positive stain. Renal cell carcinomas that exhibited NSE and/or CD56 had a poorer prognosis. [24].

## Conclusions

Incidental introperative discovery of a neuroendocine adenocarcinoma is a rare event that may arouse important diagnostic issues and therapeutic decisions. Association with chronic alcoholic pancreatitis is also rare and not fully understood. Histopathology and particularly immunohistochemical stains remain crucial for the diagnosis in respect of malignancy and prognosis.

### Consent

Written informed consent was obtained from the patient for publication of this Case Report and any accompanying images. A copy of the written consent is available for review by the Editor-in-Chief of this journal.

## Abbreviations

LSAB: Enzyme-labeled streptavidin-biotin; WHO: World Health Organisation; MIB-1: Marker for cell proliferation, directed against the nuclear Ki-67 antigen; NSE: Neuron specific enolase; CD56: Equivalent NCAM (neural cell adhesion molecul); CgA: Chromogranin A.

## Competing interests

The authors declare that they have no competing interests.

## Authors' contributions

VS and SR contributed to aquisition of data, conception and drafting of manuscript. MG performed histologic and immunohistochemic analysis and provided results. EP reviewed the manuscript and gave final approval of the version to be published.

## References

[B1] Rampurwala MM Kumar A Kannan S Kowalczyk P Khera: Non-Functioning Pancreatic Neuroendocrine Tumors-A Case Report and Review of Literature. J Gastrointest Canc 2010 Oct 23, [Epub ahead of print] 10.1007/s12029-010-9223-3 20967573

[B2] Hill JS McPhee JT McDade TP Zhou Z Sullivan ME Whalen GF Tseng JF Pancreatic neuroendocrine tumors: the impact of surgical resection on survival Cancer 2009 115 4 741 751 10.1002/cncr.24065 19130464

[B3] Modlin IM Oberg K Chung DC Jensen RT de Herder WW Thakker RV Caplin M Delle Fave G Kaltsas GA Krenning EP Moss SF Nilsson O Rindi G Salazar R Ruszniewski P Sundin A Gastroenteropancreatic neuroendocrine tumors Lancet Oncol 2008 9 61 72 10.1016/S1470-2045(07)70410-2 18177818

[B4] Kimura W Kuroda A Morioka Y Clinical pathology of endocrine tumors of the pancreas. Analysis of autopsy cases Dig Dis Sci 1991 36 933 942 10.1007/BF01297144 2070707

[B5] Shrikhande S Kleeff J Zimmermann A Friess H Büchler MW Co-existent chronic pancreatitis and pancreatic neuroendocrine tumor. Case report and review of the literature Pancreatology 2001 1 2 117 122 10.1159/000055803 12120189

[B6] Davies K Conlon KC Neuroendocrine tumors of the Pancreas Curr Gastroenterol Rep 2009 11 119 127 10.1007/s11894-009-0019-1 19281699

[B7] Klöppel G Heitz PU Pancreatic endocrine tumours Pathol Res Pract 1988 183 155 168 10.1016/S0344-0338(88)80043-8 2898775

[B8] Ehehalt F Saeger HD Schmidt CM Grützmann R Neuroendocrine tumors of the pancreas Oncologist 2009 14 456 467 10.1634/theoncologist.2008-0259 19411317

[B9] Ito T Tanaka M Sasano H Osamura YR Sasaki I Kimura W Takano K Obara T Ishibashi M Nakao K Doi R Shimatsu A Nishida T Komoto I Hirata Y Imamura M Kawabe K Nakamura K Preliminary results of a Japanese nationwide survey of + neuroendocrine gastrointestinal tumors J Gastroenterol 2007 42 497 500 10.1007/s00535-007-2056-6 17671766

[B10] Kazanjian KK Reber HA Hines OJ Resection of pancreatic neuroendocrine tumors. Results of 70 cases Arch Surg 2006 141 765 770 10.1001/archsurg.141.8.765 16924083

[B11] Oberg K Eriksson B Endocrine tumors of pancreas Best Pract Clin Gastroenterol 2005 19 753 781 10.1016/j.bpg.2005.06.002 16253899

[B12] Rickes S Unkrodt K Ocran K Neye H Wermke W Differentiation of neuroendocrine tumors from other pancreatic lesions by echo-enhanced power Doppler sonography and somatostatin receptor scintigraphy Pancreas 2003 26 76 81 10.1097/00006676-200301000-00013 12499921

[B13] Falconi M Bettini R Boninsegna L Crippa S Butturini G Pederzoli P Surgical strategy in the treatment of pancreatic neuroendocrine tumors JOP 2006 7 150 156 16407638

[B14] Crippa S Bassi C Salvia R Falconi M Butturini G Pederzoli P Enucleation of pancreatic neoplasms Br J Surg 2007 94 1254 1259 10.1002/bjs.5833 17583892

[B15] Hochwald SN Zee S Conlon KC Prognostic factors in pancreatic endocrine neoplasms: an analysis of 136 cases with a proposal for low-grade and intermediate grade groups J Clin Oncol 2002 20 2633 2642 10.1200/JCO.2002.10.030 12039924

[B16] Couvelard A Sauvanet A Gastroenteropancreatic neuroendocrine tumors: indications for and pitfalls of frozen section examination Virchows Arch 2004 53 441 448 10.1007/s00428-008-0678-6 18839209

[B17] Massironi S Sciola V Peracchi M Ciafardini C Spampatti MP Conte D Neuroendocrine tumors of the gastro-entero-pancreatic system World J Gastroenterol 2008 14 35 5377 5384 10.3748/wjg.14.5377 18803349 PMC2744160

[B18] Ong SL Garcea G Pollard CA Furness PN Steward WP Rajesh A Spencer L Lloyd DM Berry DP Dennison AR A fuller understanding of pancreatic neuroendocrine tumors combined with aggressive management improves outcome Pancreatology 2009 9 583 600 10.1159/000212085 19657214

[B19] Simpson WF Adams DB Metcalf JS Anderson MC Nonfunctioning Pancreatic Neuroendocrine Tumors Presenting as Pancreatitis: Report of Four Cases Pancreas 1998 3 2 223 231 3375232 10.1097/00006676-198804000-00019

[B20] Mao C Howard JM Pancreatitis ssociated with neuroendocrine (islet cell) tumors of the pancreas Am J Surg 1996 171 6 562 564 10.1016/S0002-9610(96)00032-3 8678200

[B21] Bloomstone M Durkin A Yang I Rojiani M Rosemurgy AS Enkmann S Yeatman TJ Zervos EE Identification of Molecular Markers Specific for Pancreatic Neuroendocrine Tumors by Genetic Profiling of Core Biopsies Ann Surg Oncol 2004 11 4 413 419 10.1245/ASO.2004.03.077 15070602

[B22] Bilimoria KY Talamonti MS Tomlinson JS Stewart AK Winchester DP Ko CY Bentrem DJ Pronostic score predicting survival after resection of pancreatic neuroendocrine tumors: analysis of 3851 patients Ann Surg 2008 247 3 490 500 10.1097/SLA.0b013e31815b9cae 18376195

[B23] Armah HB Parwani AV Perepletchikov AM Henry: Synchronous primary carcinoid tumor and primary adenocarcinoma arising within mature cystic teratoma of horseshoe kidney: a unique case report and review of the literature Diagn Pathol 2009 4 17 10.1186/1746-1596-4-17 19523243 PMC2704177

[B24] Vaarala MH Kauppila S Hirvikosk P Ronkainen H Soini Y Evaluation of neuroendocrine markers in renal cell carcinoma Diagn Pathol 2010 5 28 10.1186/1746-1596-5-28 20462442 PMC2876076

